# Synthesis, Characterization and Antitumor Activity of a Series of Polypyridyl Complexes

**DOI:** 10.1155/MBD.2000.343

**Published:** 2000

**Authors:** Jie Liu, Xiao-Hua Zou, Qian-Ling Zhang, Wen-Jie Mei, Jian-Zhong Liu, Liang-Nian Ji

**Affiliations:** State Key Laboratory of Ultrafast Laser Spectroscopy/Departmentof Chemistry Zhongshan University Guangzhou 510275£ China

## Abstract

A series of polypyridyl complexes have been synthesized. All polypyridyl complexes and some of the soluble ligands have been assayed for antitumor activity in vitro against the HL-60 (the human leucocytoma) cells, BEL-7402 (the human liver carcinoma) cells, KB (the human nasopharyngeal carcinoma) cells and HELA (the human adenocarcinoma of cervix) cells. The results indicate that several complexes have relative activity against different cell lines. Especially, the complexes [Co(bpy)_2_(pip)]^3+^, [Co(phen)_2_(pip)]^3+^, [Ru(bpy)_2_(pztp)]^2+^ and [Ru(pztp)_2_(bpy)]^2+^ show relative high activity against four tumor cell lines. Moreover, they are slightly more effective than cisplatin. At the concentration of 100 μg/mL, the complexes show inhibitory rate of 72∼86% for the cancer cells and have no toxicity for MDCK and Vero cells. It is indicated that these complexes can inhibit cancer cells selectively.


### SYNTHESIS, CHARACTERIZATION AND ANTITUMOR ACTIVITY OF A SERIES OF POLYPYRIDYL COMPLEXES

Jie Liu*, Xiao-Hua Zou, Qian-Ling Zhang, Wen-Jie Mei, Jian-Zhong Liu, Liang-Nian Ji*

State Key Laboratory of Ultrafast Laser Spectroscopy/Department of Chemistry, Zhongshan University, Guangzhou, 510275£¬P. R. China)

### Abstract

A series of polypyridyl complexes have been synthesized. All polypyridyl complexes and some of the soluble ligands have been assayed for antitumor activity in vitro against the HL-60 (the human leucocytoma) cells, BEL-7402 (the human liver carcinoma) cells, KB (the human nasopharyngeal carcinoma) cells and HELA (the human adenocarcinoma of cervix) cells. The results indicate that several complexes have relative activity against different cell lines. Especially, the complexes $[Co(bpy)_2(pip)]^{3+}$, $[Co(phen)_2(pip)]^{3+}$, $[Ru(bpy)_2(pztp)]^{2+}$ and $[Ru(pztp)_2(bpy)]^{2+}$ show relative high activity against four tumor cell lines. Moreover, they are slightly more effective than cisplatin. At the concentration of 100 μg/mL, the complexes show inhibitory rate of 72~86% for the cancer cells and have no toxicity for MDCK and Vero cells. It is indicated that these complexes can inhibit cancer cells selectively.

**Key words:** Polypyridyl complexes, Antitumor activity, Cytotoxicity

### Introduction

Currently, *cis*-diaminedichlorplatinum(II) (cisplatin) is one of the three most widely used antitumor drugs in the world. It is highly effective in treating testicular and ovarian cancers[1,2]. Despite its success, cisplatin has several disadvantages that include severe toxicity such as nephrotoxicity, neurotoxicity, and emetogensis. The toxic side effects of cisplatin limit the dose that can be given to patients. Another platinum-based antitumor drug, diamine[1,1-cyclobutanedicaboxylato(2-)]-platinum(II) (carboplatin), received worldwide approval and achieved routine clinical use. Carboplatin is less toxic than cisplatin and can be given at a much higher dose than cisplatin. Unfortunately, carboplatin is still only active in the same range of tumors as cisplatin and is still administered intravenously[3]. Thus, it is necessary to develop a new class of anticancer agents.

Some metal complexes containing polypyridyl ligands are known to be bound with DNA by intercalation[4~6]. However, little attention has been paid to the anticancer activity of polypyridyl metal complexes and a systematic investigation on the anticancer activity and cytotoxicity of polypyridyl complexes is rare. In fact some metal polypyridyl complexes display remarkable antibacterial and antitumor activity[7~11].

In the present paper, we report for the fist time the cytotoxic activity of a series of Ru(II) / Co(III) based polypyridyl complexes. It was expected to find a new type of potential antitumor agent from these complexes.

### Materials and methods

### *Complexes*

Synthesis of the ligand pytp[12] and the complexes $[Ru(L)_2(ohtp)]^{2+}$[13], $[Co(L)_2(ip)]^{3+}$ and $[Co(L)_2(pip)]^{3+}$ (L = bpy or phen)[14] have been described. Complexes $[Ru(bpy)_2(pytp)]^{2+}$ and $[Ru(phen)_2(pytp)]^{2+}$ were synthesized similarly to a previously described method[15].

*Synthesis of* $[Ru(bpy)_2(pytp)](ClO_4)_2$

0.048 g (0.35 mmol) of pyridine-2-carboxamide hydrazone[16] in $C_2H_5OH$ (5 $cm^3$) was added to 10 $cm^3$ of $CH_3CN$ solution of $[Ru(bpy)_2(phendione)](ClO_4)_2$[17] (0.3 mmol, 0.260 g). The mixture was refluxed at 80°C for 4 h and evaporated to dryness. The residue was dissolved in $CH_3CN$ (5 $cm^3$) and purified by column chromatography on alumina using acetonitrile/toluene (10:1) as eluent. Yield: 0.167 g, 60%. Anal. Calcd for $[Ru(bpy)_2(pytp)](ClO_4)_2$ C, 45.4; H, 3.16; N, 15.6. Found: C, 45.8; H, 3.32; N, 15.4. $^1H$

NMR (500 MHz, DMSO-$d_6$): (ppm) 9.85 (d, 1 H), 9.76 (d, 1 H), 8.99 (m, 2 H), 8.61 (t, 4 H), 8.38 (d, 1 H), 8.33 (d, 1 H), 8.19 (t, 3 H), 8.09 (t, 2 H), 8.03 (m, 2 H), 7.92 (d, 2 H), 7.77 (m, 3 H), 7.54 (t, 2 H), 7.34 (m, 2 H).

*Synthesis of [Ru(phen)$_2$(pytp)](ClO$_4$)$_2$*

This complex was synthesized in a similar manner to that described for [Ru(bpy)$_2$(pytp)](ClO$_4$)$_2$, with 0.3 mmol, 0.261 g of [Ru(phen)$_2$(phendione)](ClO$_4$)$_2$ [17] in place of [Ru(bpy)$_2$(phendione)](ClO$_4$)$_2$. Yield: 0.189 g, 65%. Anal. Calcd for [Ru(phen)$_2$(pytp)](ClO$_4$)$_2$ C, 50.7; H, 2.59; N, 15.7. Found: C, 50.9; H, 3.09; N, 15.6. $^1$H NMR (500 MHz, DMSO-$d_6$): (ppm) 9.81 (d, 1 H), 9.73 (d, 1 H), 8.98 (m, 2 H), 8.70 (d, 2 H), 8.67 (d, 2 H), 8.33 (s, 4 H), 8.30 (d, 2 H), 8.27 (d, 2 H), 8.19 (t, 1 H), 8.10 (d, 1 H), 8.08 (d, 1 H), 7.89 (m, 2 H), 7.72 (m, 5 H).

All other complexes [Ru(bpy)$_2$(dppz)]$^{2+}$, [Ru(phen)$_2$(dppz)]$^{2+}$ [18], [Ru(bpy)$_2$(pztp)]$^{2+}$ [15], [Ru(phen)$_2$(pztp)]$^{2+}$ and [Ru(pztp)$_2$(bpy)]$^{2+}$ [19] were synthesized according to literature methods. Structures of some related ligands are illustrated in Chart 1. Elemental analyses were performed on a Perkin-Elmer 240C elemental analyzer.

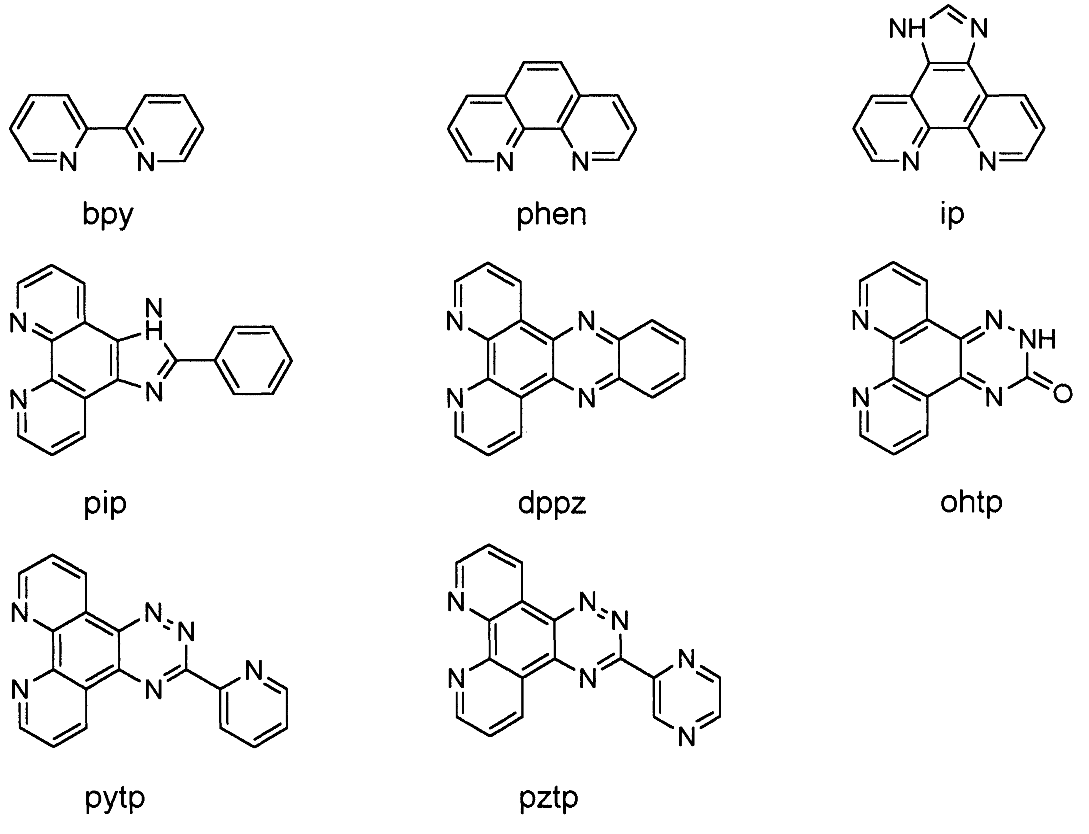

Chart 1. Structures of the polypyridyl ligands

Solvents were purified by standard techniques and were freshly distilled prior to use. Other chemicals used are analytical reagents of high purity grade.

For experiments the complexes were dissolved in freshly prepared in 10 % DMSO, and diluted to the required concentration with culture when used. The stock solutions in distilled water were stored at +4°C. The solutions in growth medium used in the experiments were prepared extempore. Cisplatin was used as a reference compound and its solutions were prepared as described above[20].

***Cells and tumor cells***

The four tumor cells strains HL-60 (the human leucocytoma) cell line, BEL-7402 (the human liver carcinoma) cell line, KB (the human nasopharyngeal carcinoma) cell line, and

HELA (the human adenocarcinoma of cervix) cell line were provided by the National Key Laboratory of Natural and Bionic Drugs, Beijing Medical University.

Madin-Darby Canine Kidney MDCK cells and Vero cells were obtained from the Chinese Academy of Military Medical Sciences.

The cells and tumor cells were cultured at 37°C as monolayers in RPMI-1640 medium (Flow Laboratories, USA) supplemented with antibiotics (penicillin and streptomycin) and 10% bovine serum. Serum concentration was reduced to 5% for growth of cells and tumor cells and for testing the complexes[21].

***Cytotoxicity assay of the compounds***

The compounds were dissolved in RPMI-1640 containing 2% Calf serum, filtered to remove germs, and diluted with cell maintenance medium to obtain the solution with different concentrations. Having formed a single layer, MDCK and Vero cells were digested by pancreatin and counted, then made into a $2x10^5$ cells/ml suspension and inoculated in the wells of flat-bottomed 96-well plastic culture tray (0.1 ml/well), thereafter incubated at 37°C in a thermotank containing 5% $CO_2$ for 24 h. After the cells had formed single layers, the original culture medium was removed and replaced with maintenance medium containing various concentrations of the compounds. After further incubation for 5-7 days, the number of the living cells was calculated by MTT staining method[22]. The maximum nontoxic concentration ($TC_0$) and the 50% toxic concentration ($TC_{50}$) were also calculated.

***Antitumor activity assay of the compounds***

The complexes and cisplatin were assayed for cytotoxicity in vitro against The four tumor cells strains HL-60 (the human leucocytoma) cell line, BEL-7402 (the human liver carcinoma) cell line, KB (the human nasopharyngeal carcinoma) cell line, and HELA (the human adenocarcinoma of cervix) cell line. The procedure for antitumor activity studies was similar to that reported earlier[23,24]. Briefly, in order to calculate the concentration of each drug that produces a 50% inhibition of cell growth ($IC_{50}$), 100 μl of cell suspension ($4x10^5$ cell/ml) were exposed to various concentrations of complexes dissolved in sterile water. After incubation periods of 72 h for all cell lines, the cell concentrations were determined both in control and in drug-treated cultures. All experiments were made in quadruplicate.

The results show that several complexes have relative activity against different cell lines.

Table 1. Cytotoxicity values of the polypyridyl complexes

| Complexes [a] | MDCK | | Vero | |
|---|---|---|---|---|
| | $TC_0$(μmol/L) | $TC_{50}$(μmol/L) | $TC_0$(μmol/L) | $TC_{50}$(μmol/L) |
| $[Co(bpy)_2(ip)]^{3+}$ | 480 | 690 | 450 | 650 |
| $[Co(bpy)_2(pip)]^{3+}$ | 420 | 630 | 430 | 620 |
| $[Co(phen)_2(pip)]^{3+}$ | 380 | 540 | 360 | 540 |
| $[Co(phen)_2(ip)]^{3+}$ | 490 | 560 | 470 | 520 |
| $[Ru(bpy)_2(dppz)]^{2+}$ | 390 | 480 | 400 | 500 |
| $[Ru(phen)_2(dppz)]^{2+}$ | 450 | 650 | 420 | 610 |
| $[Ru(bpy)_2(ohtp)]^{2+}$ | 320 | 460 | 340 | 490 |
| $[Ru(bpy)_2(pztp)]^{2+}$ | 360 | 480 | 380 | 500 |
| $[Ru(phen)_2(pztp)]^{2+}$ | 350 | 480 | 380 | 500 |
| $[Ru(pztp)_2(bpy)]^{2+}$ | 380 | 500 | 390 | 520 |
| $[Ru(bpy)_2(pytp)]^{2+}$ | 320 | 400 | 360 | 460 |
| $[Ru(phen)_2(pytp)]^{2+}$ | 300 | 410 | 350 | 480 |

[a] All the complexes are in perchlorate form except that $[Co(bpy)_2(pip)]^{3+}$ and $[Co(phen)_2(pip)]^{3+}$ are in chloride form.

### Results

All complexes are characterized by elemental analyses, UV-Vis spectroscopy and $^1H$ NMR spectroscopy and are in accordance with their proposed formula.

### *Cytotoxicity and antitumor activity of the complexes*

Microscopically according to the survival rates, the maximum nontoxic concentration ($TC_0$) and the 50% toxic concentration ($TC_{50}$) of the complexes can be calculated by Reed-Muench method, and the results are listed in Table 1.

Microscopically the MNC for complexes was determined as 300 ~ 490 μmol / L for MDCK cells and 340 ~ 470 μmol / L for Vero cells (Table 1). Additional data were found when the viability of cells was studied. The number of viable MDCK cells and Vero cultured in complexes medium was increased during the whole period of investigation as compared to that of untreated control. Moreover, the viability was significantly increased when the concentration of ACV was decreased. This is well manifested after 48 and 72 h. In contrast, all complexes tested were toxic low for MDCK and Vero cell.

Polypyridyl complexes have been tested against four tumor cell lines. They were exposed to cells for 72 h and growth inhibition was assessed using the sulforhodamine B protein staining assay[25]. The corresponding 50% inhibitor dose ($IC_{50}$) values are shown in Table 2. Cisplatin is also included for comparison.

The complexes $[Ru(phen)_2(dppz)]^{2+}$, $[Ru(bpy)_2(ohtp)]^{2+}$, $[Ru(phen)_2(pztp)]^{2+}$ and $[Ru(phen)_2(pytp)]^{2+}$ showed high $IC_{50}$ values against all tested four cell lines, indicating that they have no biological activity. The complexes $[Co(bpy)_2(pip)]^{3+}$, $[Co(phen)_2(pip)]^{3+}$, $[Ru(pztp)_2(bpy)]^{2+}$ and $[Ru(bpy)_2(pztp)]^{2+}$ have much more lower $IC_{50}$ values against HL-60 cell line. Moreover, they are slightly more effective than cisplatin. The complexes $[Co(bpy)_2(pip)]^{3+}$, $[Co(phen)_2(pip)]^{3+}$, $[Ru(pztp)_2(bpy)]^{2+}$ and $[Ru(bpy)_2(pztp)]^{2+}$ displayed similar $IC_{50}$ values (7.3, 9.4, 9.8 and 6.7 μmol/L respectively) against HL-60 cell line to cisplatin (6.0 μmol/L), indicating that the four polypyridyl complexes could be considered to have same cytotoxic activity. In addition, although the complexes $[Co(bpy)_2(pip)]^{3+}$ and $[Ru(bpy)_2(pztp)]^{2+}$ showed higher $IC_{50}$ values (8.9 and 6.9 μmol/L respectively) against KB cell line than cisplatin (1.3 μmol/L), they still exhibited good cytotoxic activity.

It is very interesting that $[Ru(bpy)_2(dppz)]^{2+}$ and its sister complex $[Ru(bpy)_2(dppz)]^{2+}$ do not show any significant inhibition against all of the tested cell lines despite that they are proven DNA intercalators and bind to DNA avidly ($K_b > 10^6$)[26]. Furthermore, being associated with DNA electrostatically[13], $[Ru(bpy)_2(ohtp)]^{2+}$ is non-active against the cell lines either. DNA binding studies suggested that the other complexes should be bound with DNA by intercalation[14,15,19], but their antitumor activities against the cell lines are diverse. For instance, $[Ru(bpy)_2(pztp)]^{2+}$ shows much more higher activity against all of the four cell lines than $[Ru(phen)_2(pztp)]^{2+}$ in spite of the similarity of their structures. The results indicate clearly that the antitumor mechanisms of these complexes should be different and the central metal ions, the ligands and the shape of the complexes should play a role in the mechanism. Moreover, since all of these polypyridyl complexes bind to DNA non-covalently, the antitumor mechanisms should also be different from that of cisplatin. Recently published data also show that the inhibition of antitumor activity by polypyridyl ruthenium or cobalt was enhanced by reducing agents and that the mechanism of the activation is similar to that for polypyridyl complexes mediated DNA damage.

### Conclusion

Twelve polypyridyl metal complexes have been synthesized and characterized. These complexes have been tested in vitro against HL-60 (the human leucocytoma) cell line, BEL-7402 (the human liver carcinoma) cell line, KB (the human nasopharyngeal carcinoma) cell line and HELA (the human adenocarcinoma of cervix) cell line. It has been found that if an appropriate polypyridyl ligand is bound to Ru(II) or Co(III), the corresponding complex could exhibit a significant antitumor effect. The present study indicates for the first time that polypyridyl metal complexes have significant cytotoxic activity. We believe our results may contribute to the development of a new class of antitumor agents.

Table 2. 72 h $IC_{50}$ values obtained for the polypyridyl complexes and cisplatin against different tumor cells lines

| Complexes [a] | $IC_{50}$(μmol/L) [b] | | | |
|---|---|---|---|---|
| | HL-60 | BEL-7402 | KB | HELA |
| [Co(bpy)$_2$(ip)]$^{3+}$ | 57.8$^{*}$ | >100$^{*}$ | 18.9$^{**}$ | 10.4$^{**}$ |
| [Co(bpy)$_2$(pip)]$^{3+}$ | 7.3$^{**}$ | 13.2$^{**}$ | 8.9$^{**}$ | 12.6$^{**}$ |
| [Co(phen)$_2$(pip)]$^{3+}$ | 9.4$^{**}$ | 34.6 | 54.6$^{*}$ | 32.5$^{*}$ |
| [Co(phen)$_2$(ip)]$^{3+}$ | 92.6$^{*}$ | - | >100$^{*}$ | >100$^{*}$ |
| [Ru(bpy)$_2$(dppz)]$^{2+}$ | 92$^{*}$ | - | >100$^{*}$ | >100$^{*}$ |
| [Ru(phen)$_2$(dppz)]$^{2+}$ | -[c] | - | - | - |
| [Ru(bpy)$_2$(ohtp)]$^{2+}$ | - | - | - | - |
| [Ru(bpy)$_2$(pztp)]$^{2+}$ | 6.7$^{**}$ | 12.6$^{**}$ | 6.9$^{**}$ | 12.3$^{**}$ |
| [Ru(phen)$_2$(pztp)]$^{2+}$ | - | - | >100$^{*}$ | >100$^{*}$ |
| [Ru(pztp)$_2$(bpy)]$^{2+}$ | 9.8$^{**}$ | 6.5$^{**}$ | 12.3$^{**}$ | 24.5$^{**}$ |
| [Ru(bpy)$_2$(pytp)]$^{2+}$ | 65.4$^{*}$ | 9.6$^{**}$ | 14.6$^{**}$ | 6.8$^{**}$ |
| [Ru(phen)$_2$(pytp)]$^{2+}$ | 98.6$^{*}$ | >100$^{*}$ | >100$^{*}$ | >100$^{*}$ |
| Cisplatin | 6.5 | 7.7 | 1.3 | 6.9 |

*a*. All the complexes are in perchlorate form except that [Co(bpy)$_2$(pip)]$^{3+}$ and [Co(phen)$_2$(pip)]$^{3+}$ are in chloride form. *b*. The 50% inhibitory concentration ($IC_{50}$) is defined as concentration that suppresses tumor cells by 50%. *c*. The inhibitory rate against tumor cells less than 50% at testing concentration of the complexes.

$^{*}p<0.5$ $^{**}p<0.05$

### Acknowledgments

We are grateful to the National Natural Science Foundation of China, Institute of Medicinal Biotechnology, Peking Union Medical College, Chinese Academy of Medical Sciences in Beijing and National filtration center of new drugs.

### References

1. Rosenberg, B. *in Metal Ions in Biological Systems,* Sigel, H. Ed. Marcel Dekker, New York, 1980,Vol. **11**, pp. 127-196.
2. Reedijk, J. *Inorg. Chim. Acta.* 1992, **198,** 873-881.
3. Wong, E., Giandomenico, C. M. *Chem. Rev.* 1999, **99,** 2451-2466.
4. Liu, J.-G., Ye, B.-H., Li, H., Zhen, Q.-X., Ji, L.-N. *J. Inorg. Biochem.* 1999, **76,** 265-271.
5. Liu, J.-G., Ye, B.-H., Li, H., Zhen, Q.-X., Zou, X.-H., Ji, L.-N. *J. Biol. Inorg. Chem.* 2000, **5,** 119-128.
6. Zhen, Q.-X., Ye, B.-H., Zhang, Q.-L., Liu, J.-G., Ji, L.-N. *J. Inorg. Biochem.* 1999, **76,** 47-53.
7. Vilchez, F. G., Vilaplana, R., Blasco, G., Messori, L. *J. Inorg. Biochem.* 1998, **71,** 45-52.
8. Birch, N. J. *Chem. Rev.* 1999, **99,** 2659-2682.
9. Sullivan, B. P., Salmon, D. J., Meyer, T. J., *Inorg. Chem.* 1978, **17,** 3334-3341.
10. Fruhauf, S., Zeller, W. J. *Cancer Res.* 1991, **51,** 2943-2951.
11. Kratz, F., Schutte, M. T. *Cancer J.* 1998, **11**, 60-67.
12. Zou, X.-H., Cai, J.-W., Feng, X.-L., Hu, X.-P., Yang, G., Zhang, H., Ji, L.-N. *Transition Metal Chem.* Accepted for publication.
13. Zou, X.-H., Li, H., Yang, G., Deng, H., Liu, J., Li, R.-H., Zhang, Q.-L., Chao, H., Xiong, Y., Ji, L.-N. *Inorg. Chem.* Submitted.
14. Zhang, Q.-L., Liu, J.-G., Chao, H., Ji, L.-N. *J. Inorg. Biochem.* In press.
15. Zou, X.-H., Ye, B.-H., Li, H., Liu, J.-G., Xiong, Y., Ji, L.-N. *J. Chem. Soc. Dalton Trans.* 1999, 1423-1428.
16. Case, F. H. *J. Org. Chem.* 1965, **30,** 931-933.
17. Goss, C. A., Abruna, H. D. *Inorg. Chem.* 1985, **24,** 4263-4267.

18. Amouyal, E., Homsi, A., Chambron, J. C., Sauvage, J. P. *J. Chem. Soc. Dalton Trans.* 1990, 1841-1845.
19. Zou, X.-H., Ye, B.-H., Li, H., Zhang, Q.-L., Chao, H., Liu, J.-G., Ji, L.-N. *J. Biological Inorg. Chem.* 2001, **6,** 143-150.
20. Butour, J.L., Wimmer, S., Wimmer, F., Castan, P. *Chem. Biol. Interact.* 1997, **104,** 165-178.
21. Liu, J., Mei, W.-J., Li, Y.-G., Wang, E.-B., Ji, L.-N., Tao, P.-Z. *Antiviral Chem. Chemother.* 2000, **11,** 331-336.
22. Pauwele, R. Balzarini, J., Baba, M., Snoeck, R., Schols, D., Herdewijn, P., Desmyter, J. Declercq, E. *J. Virol. Methods.* 1988, **20,** 309-321.
23. Zhao, G-H., Lin, H-K., Zhu S-R., Sun, H-W., Chen, Y-T. *J. Inorg. Biochem.* 1998, **70,** 219-226.
24. Hill, C. L., Weeks, M. S., Schinazi, R. F. *J. Med. Chem.* 1990, **33,** 2767-2772.
25. Skehan, P., Storeng, R., Scudiero, D., Monks, A., McMahon, J., Vistica, D., Warren, J., Bokesch, H., Kennedy, S., Boyd, M. R. *J. Natl. Cancer Inst.* 1990, **82,** 1107-1112.
26. Friedman, A. E., Chambron, J. C., Sauvage, J. P., Turro, N. J., Baeton, J. K. *J. Am. Chem. Soc.* 1990, **112,** 4960-4961.

**Received: February 21, 2001 – Accepted: February 28, 2001 – Received in revised camera-ready format: March 1, 2001**

